# Civet latrines in three habitats of a coffee dominated landscape of the Western Ghats biodiversity hotspot

**DOI:** 10.1038/s41598-023-50193-2

**Published:** 2023-12-20

**Authors:** Palatty Allesh Sinu, P. P. Fahira, T. P. Rajesh, Gopika Viswan, K. Manoj, M. Hariraveendra, Thomas Jose

**Affiliations:** Central University of Kerala, Periya, Kasaragod, Kerala 671316 India

**Keywords:** Ecology, Biodiversity

## Abstract

Civets are frugivorous animals in the Order Carnivora. They are relatively less shy towards people and anthropogenic habitats. It has been reported that the civets’ preference of defecating in open sites enable them to be important seed dispersers of degraded forests and urban ecosystems of Asia and Africa. We surveyed for scats of palm civet (*Paradoxurus hermaphroditus*) in forest fragments of sacred groves (closed), coffee plantations (partly closed) and home gardens (relatively open) during the fruit ripening period of Coffee and *Caryota urens* – the two preferred fruits of civet – to report the microhabitat characteristics and seed composition of civet latrines. The microhabitat of each scat position – whether on or off the ground and the shade type – was recorded. The scat analysis showed the presence of 4234 seeds belonging to coffee (90.2%), *C. urens* (9.7%), and an anonymous Rubiacea species (0.10%) in a total of 105 scats collected from coffee plantations (55), home gardens (5), and sacred groves (45). The number of scats sampled from the three habitats was different, but not the number of seeds per scat. Overall, the number of scats increased with the canopy cover, but the trend was different for different habitats. In home garden and coffee plantations, it decreased, but in sacred groves, it increased with the canopy cover. The number of scats sampled above the ground – on tree branches, logs and built-up structures– was more than that was on the ground. The findings contradict the general belief that the civet latrines occur more in open areas than the shaded areas. Because the civet latrines are seen more above ground than on the ground, their efficiency as seed dispersal agent may be examined critically in different contexts.

## Introduction

The small mammals in Herpestidae (Mongooses), Mustelidae (Badgers, Martens, and Weasels), Procyonidae (Coatis and Kinkajous), and Viverridae (Civets), though belong to the Order Carnivora, have fruits and other plant parts as part of their diet. Among them, the Civets in Old-world- and Afro-tropics, and Kinkajous in Neotropics have fruits as a major portion of their diet (> 90%) throughout the year^1^. They are abundant in their respective geographical ranges and less shy towards peoples and anthropogenic habitats, such as plantations, fragmented and degraded forests, human settlements, and urban ecosystems. Therefore, IUCN has listed most of them as the least concerned species and important dispersers of seeds of degraded, fragmented, urbanized forests and other disturbed habitats^1,2^.

The frugivores, in order to benefit plants as dispersal agents, must direct seeds to favorable microclimates that can offer good biotic and abiotic conditions for recruitment, less competition from recruits and less mortality from seed predators and seedling herbivores^3–7^. Therefore, the defecation habit, habitat and microhabitat are critical for assessing the efficiency of frugivores as dispersal agents and to predict the fate of animal-dispersed seeds^8–13^.

Like other small carnivorous mammals, civets also habitually defecate in conspicuous places^14,15^. They cross and even opt for open habitats for foraging and defecation^2,8,16^. Therefore, they can facilitate plants colonizing and creating communities in unpredictable conspicuous and unique habitats like the frugivorous bats – another disturbance-tolerant seed dispersal agent – do^17^. Nevertheless, very few studies have studied the habitat and microhabitat characteristics of civet latrines (hereafter, civetrines).

Corlett^8,18^ and Nakashima et al.^15,16^ report that civets as a taxonomic group prefer open habitat for defecation. However, Mudappa et al.^1^ and Chakravarthy and Ratnam^19^ have sampled civet scats on tree branches, fallen logs and ground, but under the closed canopy. Nakashima et al.^15^ found disproportionately large number of civet scats of common palm civet on ground along river banks, abandoned trails, tree fall gaps and rainwater runoffs. They showed that the seeds of *Leea aculeata* in the scats deposited in tree fall gaps and stream beds germinated well, but poorly germinated in other microhabitats. It is likely that the fruit harvesting site (trees or fallen fruits on ground) can predict defecation sites^19^. In a tropical site, the common palm civet foraged fruits of *Vitex glabrata* and *Prunus ceylonica* respectively from the tree branches and forest ground, and subsequently deposited the seeds there itself^19^.

In the present study, the civet scats of common palm civet (*Paradoxurus hermaphroditus*) were sampled from three habitats that vary by the shade cover – tropical evergreen forests of sacred groves (closed habitat), coffee plantations (partially closed habitat) and home gardens (open habitat)– during the fruiting season of Coffee and *C. urens* – the two favorite diet of common palm civets to report the microhabitat characteristics and seed composition of civet latrines. We went with the hypothesis that the civet latrines would be encountered more in less-shaded or open habitats.

## Material and methods

### Study area

The study was conducted in a coffee-dominated landscape of Kodagu district of Karnataka state in south India. The study site is characterized by a mosaic of coffee plantations, sacred groves and home gardens and locates on the eastern slope of the Western Ghats biodiversity hotspot between the latitudes of 11° 57′ N and 12° 48′ N and between the longitudes of 74°55′ E to 76° 00′ E at an altitudinal range of 800–1500 m asl. The district has an area of 4102 square km; about 35% of this area is under the cover of coffee, 50% of the area is under the cover of forests including sacred groves, and the remaining area is under home gardens and rice cultivation.

The sampling was carried out in Virajpet taluk of Kodagu district (12° 00′–12° 29′ N and 75° 39′–76° 33′ E) during November 2019–February 2020. The coffee plantations selected for sampling were small holdings (5–10 ha) and had 80% or above of its shade trees belong to native trees. The average GBH of shade trees on coffee plantations was 89 ± 18 cm. It has an average shade cover of 38 ± 5% (mean ± SD). The shade tree density of coffee plantations of Virajpet taluk ranges between 248 and 567 per ha^20^. Sacred groves are characterized by the secondary forests with a dense vegetation of many small trees (153–1624 per ha), several big trees (8–218 per ha) and a relatively closed canopy (83 ± 27%). The home gardens had sparse vegetation of several big trees (42–129 trees per ha) of mango, jackfruit, jamun, avocado and so on. It has a relatively open canopy (11.5 ± 14.7%). Please refer Sinu et al.^21^ and Prashanth Ballullaya et al.^22^ for more details of coffee plantations and sacred groves of the present study sites.

### Sampling methods

We selected eleven sites for the present study, which located apart by an average distance of about 21 km. In each site, we selected a coffee plantation, a sacred grove, and a home garden. The latter two habitats shared their boundaries with the coffee plantations. In each habitat, we laid three parallel belt transects of 250 × 10 m each, which located 300 m apart from each other. Together, we had 66 transects in coffee, and 33 each in sacred grove and home garden. Before the sites were selected for the present study, we ensured that the farmers had not collected civet scats for harvesting coffee seeds to produce “civet coffee”. In each transect, the canopy cover was visually assessed following Bellow & Nair^23^ at three points, and the mean was used.

In each transect, two researchers walked at a constant pace (approx. 1 km/h) on opposite direction twice, and searched ground, fallen trees, man-made structures, and tree branches up to a height of eight meters for civet scats. Whenever a scat was encountered, it was identified to the civet scat based on the nature and size of the scat^1,19^. All the scats we encountered were intact, cylindrical, non-pungent and non-coiled, and identified to the scat of Common-Palm Civet (*P. hermaphroditus*). The study site also has another species of civet – the Small Indian-Civet (*Viverricula indica*) – but are spotted rarely in the region. They have a black and white ringed tail. The position of each scat was marked and visually assessed the local canopy cover and given a relative rank of closure level on the scale of 0% (open) -100% (closed). If a civet scat was sampled directly under the shade of coffee plants, the canopy cover of that point was assessed.

The civet scat positions were identified as one of the follows: (1) man-made structures, such as concrete or brick and mortar compound wall, tile thatches of buildings, premises of temples, and other built-up structures (hereafter, man-made structures); (2) tree branches and fallen tree trunks (hereafter, fallen trees); and (3) ground. The scats were carried in separate paper covers to the field camp, where the seeds deposited in the scats were retrieved, counted, and identified to species or morphospecies. Scats having more than one seed species were assigned to the respective seed categories during analysis.

### Statistical analyses

The three transects were pooled to find out mean number of scats and mean canopy cover. First, the canopy cover of three habitats was compared using one-way ANOVA test. Generalized linear models were used to test for the effect of habitat on number of scats and the number of seeds/scat. In the models, the habitat was used as the independent variable. Because the total number of transects in coffee (66 transects) was double the number of the other two habitats (33 each), mean number of scats per transect was used as the dependent variable in the model that was used to study the effect of habitat on scat numbers. To study the effect of habitat on the number of seeds deposited along with the scats, the mean number of seeds per scat was used as the dependent variable. We repeated this analysis for each seed species – Coffee and *Caryota urens*. In the model, negative binomial error was fitted as the type of distribution, and all the final fitted models were checked for overdispersion using the R package DHARMa. The significance of the final GLM model was examined using one-way ANOVA available in the R package, *car*.

To examine if the scat and seed deposition were driven by the canopy cover, the number of scats and seeds/scat were modeled on the canopy cover of the transects in the linear models. Further, analysis of covariance was used to examine if the interaction between canopy cover and habitat influenced the scat and seed deposition. In this model, the interaction of habitat and the canopy cover was used as the independent variable and the number of scats and number of seeds/scat were used as the dependent variables.

Chi-square test was used to see if the number of scats sampled in the three microhabitats – man-made structures, tree branches and logs, and ground – was similar. Later, one-way ANOVA was used to compare the numbers of coffee and *C. urens* seeds deposited in the scats of three microhabitats. In the model, the number of seeds/scat was used as the response variable, and microhabitat as the independent variable. Although it is likely that the seeds deposited above the ground could be secondarily dispersed to ground or moved by caching rodents, the scats we sampled were intact and had no signs of secondary dispersal or post-dispersal predation of seeds by rodents. Therefore, the scats deposited on trees and man-made structures were subsequently grouped into ‘above the ground scats’ as the fate of the seeds of those scats could be the same, and compared their number with that were deposited on ground. All data analyses were performed in R version 3.2.3.

## Results

The three habitats were different on the shade cover; the sacred groves were relatively closed (83 ± 27% SD), home gardens were relatively open (11.5 ± 14.7%), and coffee plantations were partially closed (34 ± 26.7%) (F_2,30_ = 1420, p < 0.00005). Over the entire study, 32% (N = 132 transects) of the transects yielded civet scats (Table 1). The proportion of transects sampled civet scats was different for the three habitats (prop.test: χ^2^ = 15.7, df = 2, p = 0.0004). A total of 105 civet scats was sampled from the whole study. This was distributed as 55 (coffee plantation), 45 (sacred grove), and 5 (home garden) scats for the three habitats. The number of scats/transect in the three habitats – 1.17 (coffee plantation), 1.45 (sacred grove), and 0.15 (home garden) – was different (F_2,30_ = 4.85, p = 0.01).

**Table 1:** Summary statistics of civet scats sampled in three habitats.

Variables	Habitat		
	Home garden	Coffee plantation	Sacred grove
N_transects_	33	66	33
Transects sampled scats (%)	9	32	55
Sum number of scats	3	55	47
N_Scats/transect_ (mean ± SE)	0.15 ± 0.08	0.88 ± 0.21	1.45 ± 0.35
Sum Coffee seeds	169	1870	1780
N_Coffee seeds/scat_ (mean ± SE)	42.3 ± 16.9 (4)	41.6 ± 2.7 (45)	53.9 ± 4.7 (33)
Sum *Caryota urens* seeds	18	177	215
N_Caryota seeds/scat_ (mean ± SE)	18 (1)	16.09 ± 2.78 (11)	14.3 ± 1.8 (15)
N_Scats with >1 seed species_	–	3	3
N_Scats with exclusive Coffee seeds_	2	42	32
N_Scats with exclusive Caryota seeds_	1	10	12

Wherever relevant, the percent encounters are given in parentheses.

All the 105 scats sampled in the present study had seeds of one or more species. They together deposited 4234 seeds belonging to three plant species – Coffee (90%), *C. urens* (9.7%) and an anonymous Rubiaceae species (0.3%) (Table 1). The share of seeds moved to the three habitats – 49% (coffee plantation), 47% (sacred grove), and 4% (home garden) – was proportionate to the number of scats sampled in the three habitats. Ninety-nine scats had only one type of seed, either Coffee (76 scats) or *C. urens* (23 scats); six scats had seeds of more than one species (Table 1). Among these, four scats had Coffee and *C. urens* seeds and two scats had Coffee and the anonymous Rubiaceae seeds. The number of overall seeds collected per scat – 37.31 (± 2.63, N = 55 coffee plantation), 37.4 (± 13.99, N = 5 home garden) and 44.3 (± 4.3, N = 45 sacred grove) – was not different among habitats (F_2,102_ = 1.09, p = 0.34). The number of Coffee seeds (F_2,79_ = 2.83, p = 0.06) and *C. urens* seeds collected in the scats of the three habitats was also not different (F_2,24_ = 0.22, p = 0.8).

**Figure 1:**
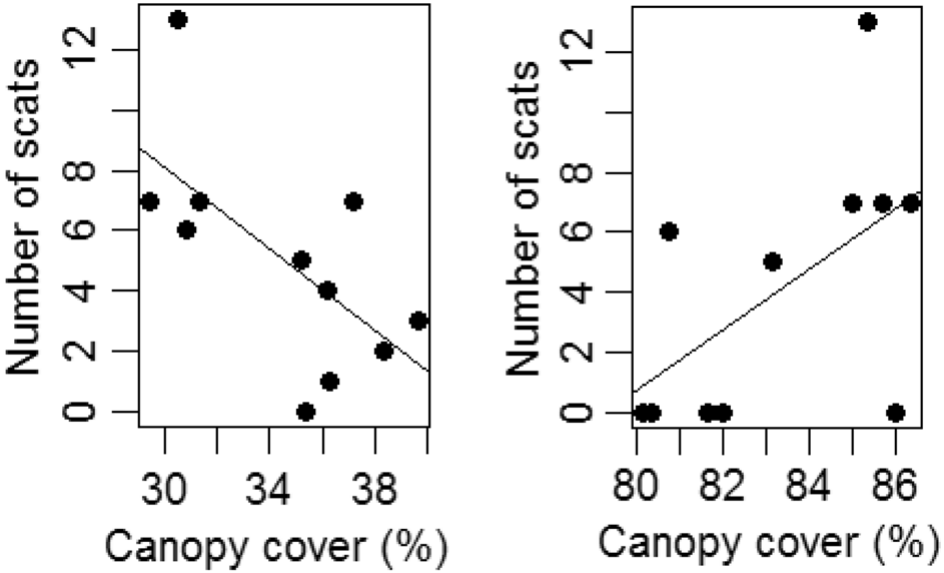
The relationship between canopy cover and number of scats. The number of scats decreased with the canopy cover in coffee plantations (left), but increased with the canopy cover in sacred groves (right).

In the habitats-pooled data, the number of scats collected per transect increased with the canopy cover of the transects (R^2^ = 0.17, F_1,31_ = 7.76, p = 0.01), but no relationship existed between canopy cover and number of seeds in scats (F_1,15_ = 0.11, p = 0.7). The interaction between habitat and canopy cover was significant for the number of scats (F_2,27_ = 7.13, p = 0.003) and the number of seeds/scat (F_1,12_ = 9.5, p = 0.009). The number of scats increased marginally with the canopy cover in sacred groves (R^2^ adjusted = 0.27, F_1,9_ = 4.74, p = 0.057), but decreased with the canopy cover in coffee plantations (R^2^ = 0.28, F_1,9_ = 4.9, p = 0.053) and home gardens (R^2^ = 0.28, F_1,9_ = 4.9, p = 0.053) (Fig. 1). Despite only weakly significant, the number of seeds in scats increased with canopy cover in sacred grove (R^2^ = 0.63, F_1,4_ = 6.8, p = 0.06), but decreased with canopy cover in coffee plantation (R^2^ = 0.33, F_1,8_ = 3.9, p = 0.08).

**Figure 2:**
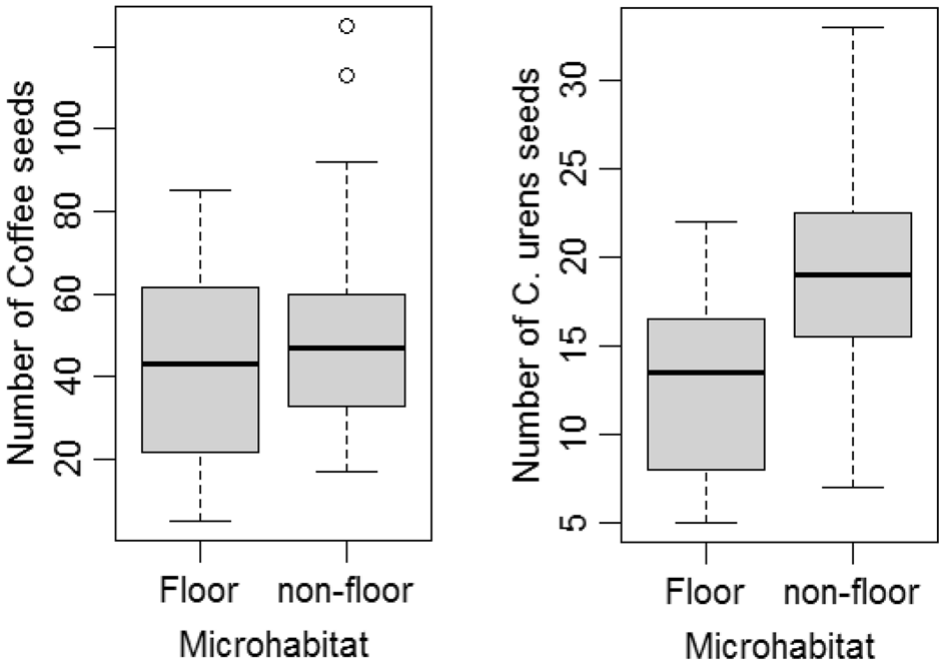
The number of Coffee and *C. urens* seeds in the civet scats deposited on and off the ground of Coffee-sacred grove-home garden matrix landscape of the Western Ghats biodiversity hotspot.

Thirty-nine scats were sampled on ground and 33 scats each were sampled on man-made structures and tree branches and fallen logs. The difference in the number of scats sampled in the three microhabitats was not significant (χ^2^ = 0.68, df = 2, p = 0.7). The numbers of coffee seeds (F_2,79_ = 1.21, p = 0.30) and *C. urens* seeds (F_2,20_ = 2.98, p = 0.07) deposited in the three microhabitats were also not different (Table 2). However, when the two above-ground microhabitats were pooled to compare the number of scats deposited on and off the ground, we collected significantly more number of scats (χ^2^ = 6.94, df = 1, p = 0.008), in particular the Coffee seeds-containing scats (χ^2^ = 4.88, df = 1, p = 0.027), off the ground than on the ground. However, the coffee seeds/scat deposited on and off the ground was not different (F_1,80_ = 2.24, p = 0.14). The number of palm seeds-containing scats on and off the ground was not different (χ^2^ = 2.13, df = 1, p = 0.14), but the number of palm seeds in scats deposited on and off the ground was significantly different (F_1,21_ = 5.43, p = 0.03) (Fig. 2).

**Table 2:** Number of scats and number of Coffee and *C. urens* seeds per scat in three microhabitats.

	Built-up structures	On ground	On tree
Total N_scats_	33 (31.4%)	39 (37.1%)	33 (31.4%)
Coffee seeds/scat	51.2 ± 5.2 (29)	42 ± 4.1 (31)	48.2 ± 3.8 (22)
*C. urens* seeds/scat	21.3 ± 4 (4)	12.9 ± 2 (8)	18.4 ± 1.8 (11)

The non-percent numbers in parentheses are the number of scats.

## Discussion

Studies have reported that the frugivorous small carnivores like civets and coatis are important seed dispersal agents of tropics. They disperse seeds through defecation. Therefore, an information of their habitat preference for defecation is fundamental to assess their efficiency as seed dispersal agents of tropical plants they associate with for frugivory. Studies have suggested that civets prefer open habitats and off-ground microsites for defecation^3,15,16,18,19^. We surveyed scats of Common Palm-Civet (*P. hermaphroditus*) during the fruiting season of coffee and *C. urens* – the two staple fruits of the civet in the study site ^1^– in three habitats of different levels of shade and physical structures to report the habitat characteristics of civet latrines. Different levels of canopy closure in the three habitats allowed us to understand if shade is a crucial factor of civet latrines. Different substrates available in the three habitats ranging from ground to thatches of buildings, and from fallen tree trunks to tree branches allowed us to understand microhabitat preferences of the civets. We used pre-determined randomly-placed transects for sampling scats in the present study, which returned 105 scats –at par to the number of seeds sampled by similar studies in other tropical parts of Asia^15,16,19^.

Although the coffee plantations were the major fruit source habitat, the number of scats sampled in coffee plantations and neighboring sacred groves were similar. The number of seeds moved in the scats to the three habitats was also similar. The findings suggest that the civets may have a preference for closed habitats for defecation in general, contradicting some previous studies^3,15,16,18^. In this study, greater number of scats was collected in sacred groves, the closed secondary forests, and least number of scats was collected in home gardens, a relatively open habitat. Most of the studies that investigated civet latrines of *P. hermaphroditus* were carried out in degraded forests or urbanized anthropogenic landscapes and are in agreement that the civets prefer open habitats for defecation^1,2,15,16,24–31^. We found that the canopy cover had a differential effect on civet latrines in open and closed habitats. In closed habitat, the number of scats increased, albeit weakly, with the canopy cover; in open habitats, the number of scats decreased, albeit weakly, with the canopy cover. Therefore, it is likely that the civets prefer open sites in disturbed environments and closed sites in shaded forest landscapes for defecation.

Another common perception on civet latrines is that they are encountered more in certain conspicuous open sites^1,8,15,18^. Nakashima et al.^15^ collected *P. hermaphroditus* scats on ground along the trails, rainwater runoffs, and river banks, but under open canopy in an early successional forest habitat in Sabah, Malaysia. Mudappa et al.^1^ encountered civet scats mostly on fallen tree logs in a closed forest of southern Western Ghats. Chakravarthy and Ratnam^19^ suggested that civet latrines of *P. hermaphroditus* can be random and on tree canopy and ground. Our results agree to Chakravarthy & Ratnam^19^ that the civet latrines of *P. hermaphroditus* can be random and on tree canopy, fallen logs of trees, and ground. The number of scats sampled off the ground was more than on the ground.

Chakravarthy & Ratnam^19^ found that the civet scats are distributed at two different vertical strata for two tree species – *Vitex glabrata* and *Prunus zeylanica* – in a forest habitat, which they have attributed to the stratum from where the fruits have been foraged. The seeds of fruits that have been harvested from ground – *P. zeylanica* – were moved predominantly to the ground, while the seeds of fruits that have been harvested from canopy – *V. glabrata* – were moved predominantly to the tree branches. Nakashima et al.^15,16^, however, found that the scat spatial distribution is not determined by the fruits the civets consume. Although two seed species were encountered in the civet scats of the present study – Coffee and *C. urens* – no contrasting pattern of spatial distribution of scats based on the seed species was noticed. But it may be noted that the coffee seeds-containing scats were collected significantly more off the ground than on the ground. The number of *C. urens* seeds-containing scats was similar on and off the ground.

Although the fruit size (1.9 cm (*C. urens*) vs. 1.8 cm (Coffee)) and seed size (1.5 cm (*C. urens*) vs. 1.3 cm (Coffee)) of Coffee and *C. urens* were similar, their seed count in the scats was highly different. A simple explanation for this variation is that these two plants have different abundances in the study site (personal observation). While coffee plants are abundant in the plantations (over 200 plants/ha), *C. urens* palms are sparsely distributed in coffee plantations and sacred groves^32^. Therefore, the amount of fruits they offer to the civets could also be different. Alternatively, the difference in the pulp quality of the fruits of *C. urens* and Coffee could also explain different numbers of their seeds in civet scats, but studying that is beyond the scope of the present study. It is, however, unlikely that the scats with different seed composition belong to different species of civet. Like other studies have noticed^1,15,16,19^, the civets have rarely mixed fruits in their diet; about 95% of the civet scats had only one type of seed, either Coffee or *C. urens*.

The scat morphotype is unique to different species of civets and other members of Viverridae^1,19^. We used the literature^1,19^, our personal sightings during and after the period of the study, and the traditional knowledge to confirm the source of scats to *P. hermaphroditus*. The findings of the present study come from a single season, the season of Coffee and *C. urens* fruiting – two major crops of the study site – and which spanned during November to March. The distribution of civet latrines may be different in other seasons, when the fruiting of forests and other horticultural crops happen in monsoon period. This caveat can be filled by a future study in the season of monsoon.

### Implications for seed dispersal

A frugicore in order to be an efficient dispersal agent of seeds must bring viable seeds to favorable microclimates that have less chance of post-dispersal seed predation, ideal microclimate for faster germination of seeds and less competition among siblings^12,13^. Nakashima et al.^15^ found *P. hermaphroditus* as an efficient dispersal agent of *Leea articulata* as its seeds were directed to favorable microclimates – the open ground of river banks. Chakravarthy & Ratnam^19^ sampled *V. glabrata* seeds predominantly in the scats deposited off the ground, which are not favourable sites for germination, unless secondary seed dispersal agents, such as dung beetles bring the seeds down to ground. The scats we sampled were also predominantly from substrata above the ground. Both the present study and Chakravarthy & Ratnam^19^ showed that the scats predominantly contain seeds of single species, which can result greater competition among recruits. 

Studying dispersal efficiency of civets by assessing the quantity and quality of seed germination and seedling recruitment though is recommended, is beyond the scope of the present study. The findings of the present study, however, is still important as a knowledge of the niche or habitat of latrines of frugivores is fundamental to assess the efficiency of a seed dispersal agent and to model the distribution of seeds. The movement of seeds by civets to open places is beneficial for pioneer plants and the movement of seeds to shades is good for climax species, but the movement of seeds to substrata above ground including tree branches, tree logs and built-up structures in anthropogenic habitats certainly downgrade the quality of a potential dispersal agent. Our study agrees that the civet’s efficiency of a seed dispersal agent can be highly varied and driven by seed traits and habitat^15,19,32^.

## Data Availability

Data can be made available upon a genuine request e-mailed to PAS.
